# Lessons from the on-site quality audit of data transmitted to the French cystic fibrosis registry

**DOI:** 10.1186/s13023-017-0750-x

**Published:** 2018-02-08

**Authors:** Nadine Pellen, Laëtitia Guéganton, Dominique Pougheon Bertrand, Gilles Rault

**Affiliations:** 1 Fondation ildys, Roscoff, France; 2 CF Center, Fondation Ildys, Roscoff, France; 3 LEPS EA3412, Sorbonne Paris Cité University, Bobigny, France

**Keywords:** Cystic fibrosis, Registry, Quality audit, Measurement recommendations

## Abstract

**Background:**

The French Cystic Fibrosis Registry takes a census of the population of patients and records their annual data transmitted by Cystic Fibrosis Centers (CFCs). Quality of patient data has been a focus in the past years, with the implementation of automated controls before data integration. The objective was to assess, at the 14 CFCs trained in the quality improvement named *Hospital Program to Improve Outcomes and Expertise in Cystic Fibrosis (PHARE-M)*, the quality of the 2012 and 2013 data transmitted to the French Registry with respect to the rules established to obtain forced expiratory volume in 1 second (FEV1%) and anthropometric data.

**Methods:**

The clinical researcher selected 20 patients at each CFC from age ranges corresponding to different visit frequencies and measurement procedures in order to reach saturation of error causes. The control consisted in comparing source data, pulmonary function tests (PFTs), patient records, and data in the Registry.

**Results:**

The audit focused on 242 patients, 2455 consultations and 1855 PFTs. Less than 5% of data concerning weight, height, or FEV1 (L) in the patient records files had discrepancies with source data. Discrepancies on patient height between patient records and PFT files were found in 11% of cases. For one hundred and ten patients (45%), anomalies were found between the patient record and the Registry for the FEV1% and the associated anthropometric measurements mainly related to the interpretation of the selection rule of the venue corresponding to the “best spirometry in the year” and the reference standard used (local standards versus Knudson reference equations). For the 33 children in the age range of 6–17 years old (27% out of 120 children records controlled), the FEV1% value in the Registry presented an average deviation of +4.25% (min. = −9.3%; max. = +16.9%; median = 4%) with the value from the Patient record.

**Conclusions:**

This first on-site quality audit of the data transmitted to the Registry pointed out variability in the measurement process at the CFCs. The rule for selecting the data for the Registry was applied differently at some CFCs, and various local References for the FEV1% calculation were used. Avenues for improvement have been identified.

## Background

### History of the French cystic fibrosis registry

The French National Cystic Fibrosis Observatory was established in 1992. Its initial objective has evolved into a comprehensive census of the population [1], allowing it to become the French Cystic Fibrosis Registry [2] certified by the French National Committee of Rare-Diseases Registries in 2008. It falls in the group of six countries whose Registry is classified as grade A based on criteria of comprehensiveness of the census population and precedence [3]. It is funded and managed by the patient organization Vaincre la Mucoviscidose with the support of the Patient Registry Steering Committee (PRSC) including the organization’s medical & scientific directors, clinicians, demographers and epidemiologists. The objectives of the French Registry are:

- To take a comprehensive census of people with cystic fibrosis by including data on diagnosis (French Association for Screening and Prevention of Child Handicaps and CFTR-France), death (CépiDc — INSERM) and transplantation (HEGP);
- To have annual data concerning the patients followed up at healthcare centers in France (mainland France, Réunion Island, and Guadeloupe);
- To help improve knowledge of the medical and social characteristics of the population with cystic fibrosis and to assess the impact of therapeutic advances on the evolution of state of health and survival;
- To assess the socioeconomic cost of this disease in terms of treatments and management and to anticipate changes in this cost; and
- To have information to shed light for the choices of parents and patients and the strategic choices of associations and other institutional partners.

The data transmitted to the Registry by the CFC teams once per year in the annual survey, according to various procedures, concern: semi-anonymous patient identification, diagnosis of cystic fibrosis, medical follow-up, social data, long term therapies prescribed, anthropometric data, pulmonary function data, and bacteriological data. The main survey is supplemented by thematic surveys: Pregnancy, *Burkholderia cepacia*, and Enrollment in Clinical Trials. Quality of patient data has been a focus for the PRSC in the past years, leading to the increase of automated controls of completeness and consistency of data before their integration in the Registry. The Registry is used for epidemiological and socioeconomic studies. Since 2006, reports by center have been issued to compare the outcomes at each CFC to the French national averages and to the outcomes at the other anonymized centers. In 2013, the French Registry recorded 6329 patients [4], 6275 (99.1%) of whom had been seen by a CFC at least once in the year. For the first time in the history of CF in France, the number of adults exceeded the number of children or adolescents in the Registry (50.6% were adults).

### PHARE performance research project

**Fig. 1:**
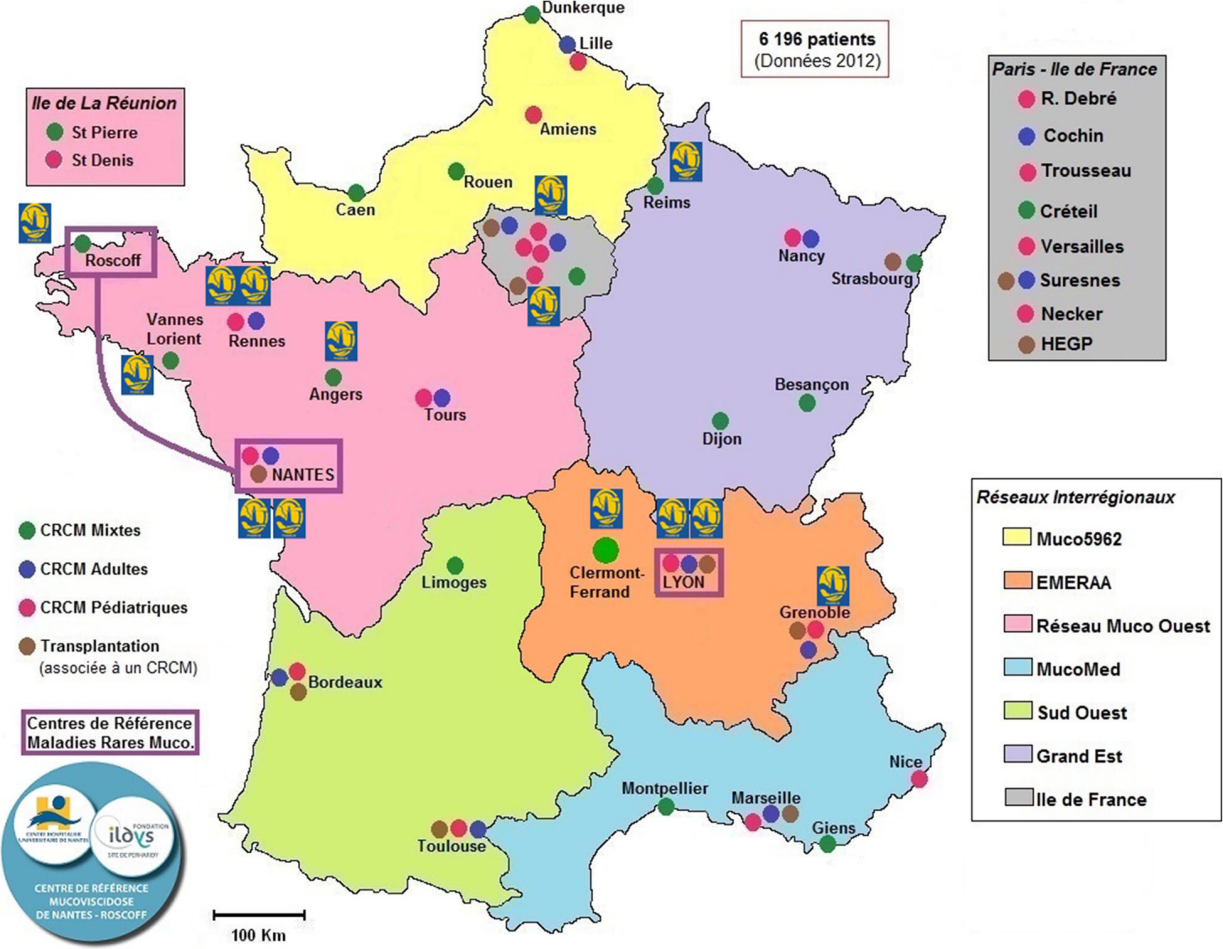
Location of the 14 CFCs involved in PHARE-M between 2011 and 2013

The PHARE-M quality improvement program (QIP) was launched in 2011–2013 in 14 CFCs willing to engage in the approach (Fig. 1). The research project, named PHARE-M Performance, funded by the French Ministry of Health in 2012, aims to assess if, in 2015, there is a measurable positive discrepancy in the trend of patient outcomes (Forced Expiratory Volume in 1 s, or FEV1, and Body Mass Index, or BMI) between patients followed up at CFCs involved in PHARE-M in 2011–2013 and patients followed up at CFCs not involved until 2015 (control group) [5]. A closed cohort was formed in 2012 for this research project including patients meeting the following criteria: genetic criteria (two CF-causing mutations [6]), uninterrupted follow-up at a CFC belonging to one of the two groups (trained or not trained in the PHARE-M), and no lung transplant. The annual Registry values for FEV1% and BMI are used as primary endpoints to determine the performance of the PHARE-M program by assessing the three-year trend (2012–2015) between the two groups of patients. The FEV1% and BMI values are calculated by the Registry software from patient’s FEV1 in liters (FEV1 L), height and weight values transmitted by the CFC. The Knudson reference equations are used to obtain the FEV1% value. Thus, best research practice led to assess the quality of the data (FEV1 L, height and weight) transmitted to the Registry to calculate FEV1% and BMI Z-score for the population enrolled in the study cohort.

Some additional hypotheses led to clarify the audit’s objectives and scope:

- The recording of reliable data in the Registry is one essential aspect of quality improvement and as such, the CFCs from the PHARE-M group must be audited so that they take actions for improvement, if necessary;
- The CFCs from the “Control” group will only be known by the end of 2015, since all those that engaged in the PHARE-M between 2013 and 2015 are excluded; thus it is difficult to audit data in this group during the course of the research project;
- The research project is not a substitute for a national Registry data quality audit, which may be decided and framed at the national level by the PRSC, should the audits conducted within the framework of this research study point to the need for such a process;
- The audits conducted on a sample of patient records at 14 CFCs should identify all the possible causes of error or, at least, all the main causes of error, in order to reach saturation of error causes.

Within the framework of the PHARE-M Performance research project, the objective was to audit, at the 14 CFCs of the PHARE-M group and on a sample of patients enrolled in the closed cohort of the study, the quality of the data transmitted to the Registry for the years 2012 and 2013, with respect to the rules established to obtain the height, weight, and forced expiratory volume in 1 second in liters (FEV1 L). A secondary objective is to assess on the sample of patients the difference on the FEV1% values between the CFC patient record and the Patient Registry, and if any, analyze the causes.

## Methods

### Patient data submitted for the audit

All data submitted for the audit were from the Registry database and hard-copy or electronic patient records and examined during on-site visits by the CRA (clinical research assistant). They include:

1. Data for patient identification by the Registry and by the CFC patient record management tool:
  ○ Patient Registry Identification No.
  ○ Initials of Last Name, First Name
  ○ Date of Birth
  ○ Gender
  ○ No. of the CFC following the patient
2. Data for enrollment in the closed cohort of the study:
  ○ Mutations in the CFTR gene
  ○ Status with respect to transplant
  ○ Status with respect to death
3. Measured data used to calculate indicators:
  ○ Date of measurement
  ○ Anthropometry: weight and height
  ○ FEV1 in L
4. Data calculated based on general population benchmarks:
  ○ FEV1 as a percentage of the expected theoretical value for age, height, and gender

### Rule for the data to be transmitted to the registry

The data transmitted annually to the Registry by the CFC teams must meet the rules established by the PRSC. Since the 2011 survey, the spirometry and anthropometry data to be transmitted to the French Registry must correspond to the visit at which the best forced expiratory volume in 1 second (FEV1) in the year has been measured, and no longer to the last visit of the patient in the year, as had been done until the 2010 survey. This rule has been worded as follows in the 2011 and subsequent questionnaire: *“Please specify the best spirometry values for the year. If there has been no spirometry: Check 'Spirometry not done' and indicate the date and the most recent anthropometry values for the year”.* Realizing the ambiguity of the wording, and given the fact that the three software used by a number of CFCs for the follow-up of CF patients automatically select the visit corresponding to the best FEV1 measured **in liters** for the patient, the Quality Control Team decided to take the following rule to designate the reference FEV1 value that should have been transmitted to the Registry: “Select the visit at which the best FEV1 L value in the year has been measured”.

### Selection of the sample of patients for the audit

The sample of patients whose data will be audited should reflect the distribution by age ranges of patients at each CFC in order to cover all the measurement procedures as defined by the international guidelines [7–9]. Thus, it must offer every opportunity to reach saturation of error causes. The audit also has to report the CFCs context in terms of amenities and local CF Patient software, including the nature (manual or automated) of the interface with the Patient Registry software, as there might be explanations regarding the quality of the data transmitted to the Registry.

The patients were selected in each CFC according to the following steps:

1. Through an email sent to the Registry administrator, the head physician at the CFC authorizes the CRA to access the patient data undergoing the quality audit.
2. The Registry administrator prints the list of patients at the CFC forming part of the population enrolled in the PHARE-M Performance research project, on which the personal data to be audited appear as they appear in the Registry.
3. The list is sent via a secure Internet connection to the head physician at the CFC and the CRA simultaneously.
4. From this list, the CRA randomly selects 20 patients, one by one, traveling through the different age ranges, until the number of 20 is reached (cf. Table I): these patients’ records will be audited in the period of time allotted to the CRA (8 h/CFC).
5. The list of patients selected is sent to the head of the CFC so that they may prepare the corresponding patient records for the CRA visit.

### Procedure for the on-site audit

1. During the on-site visit, the CRA uses the list of patients from the Registry to record the progress of the audits conducted, indicate the discrepancies in values observed, and write possible corrections that will be submitted to the CFC head physician.
2. The audit is conducted on two levels:
  ○ On the CFC internal level: comparing the PFTs and source files, the information reported in the patient record, and the information appearing in the consultation report;
  ○ On the Registry level: for each patient, assessing the data in the patient records for all their visits in the year, identifying the visit at which the best FEV1 L value for the year has been measured and comparing it with the data appearing in the Registry for that patient.
    This dual audit identifies on one hand measurement *discrepancies* and on the other hand *anomalies* for the selection of the FEV1 L value and the associated weight/height values.
3. Once the audit has been completed, the list containing the requests for correction in the Registry is printed out by the CRA and presented to the head physician for signing preceded by the statement “I acknowledge that I have read the requests for corrections to be made to the Registry. Unless I specify otherwise within a period of one month, I authorize the Registry administrator to make the necessary corrections”. A copy of the document is left to the physician on the same day.
4. At the end of the audit, a report of the visit by the CRA is given to the head physician. This report includes: an ethnographic assessment of the presentation of the patient records (classification, storage, and retention), difficulties encountered during the audit, factors having facilitated the work of data control, and recommendations for improvement.
5. After the period of 1 month, the anonymized list of patients with a request for correction is sent by the CRA to the Registry administrator to make corrections.

The CRA was in possession of a number of files equal to the number of CFCs visited: these files were circulated among the CRA, the Registry administration, and the CFC’s physician. The means ensuring personal data security focused on file storage (on an external hard disk stored in a safe at the CRA’s office) and file access audit, one per CFC (access protected by password or delivery by email accompanied by an access code delivered by SMS or telephone). The procedure for circulating data among the Registry, the Clinical Research Assistant (CRA), and the CFC received CNIL authorization [10].

## Results

The fourteen CFCs underwent the data quality audit between July 2, 2014, and June 24, 2015. This section presents the results on the two levels, the CFC level and the national Registry level. The discrepancies and anomalies found are reported by type and source of data, with the frequency of occurrence.

### Number of patient records audited

According to the 2013 Registry data, 1292 patients met the inclusion criteria in the research project population for the 14 CFCs in the PHARE-M group. Among these patients, 280 records (21%) were selected from the different age ranges according to the population distribution at the 14 CFCs (see Table 1). The population selected also had the same sex ratio as the study population.

**Table 1:** Distribution by age range of the selected patients

Age ranges	Patients selected	% of patients selected	Study population	% of study population
0–2 years	28	10	86	7
3–6 years	34	12	163	13
7–12 years	67	24	307	24
13–17 years	52	19	258	20
18–25 years	77	27	328	25
26–35 years	15	5	105	8
>35 years	7	3	45	3
Total	280	100%	1292	100%

For 2012, 13 patient records (5%) could not be consulted because they were archived off the CFC premises and could not be accessed within the period of time allotted for the visit. For 2013, seven records (2.5%) could not be found. Twenty-five available records could not be audited owing to a lack of time. In total, 242 records were audited (87 patients 18 years of age or older, and 155 patients under 18 years of age) (see Table 2).

**Table 2:** Distribution of the number of records audited by CFC

CFC	1	2	3	4	5	6	7	8	9	10	11	12	13	14	Total
Study population	108	86	71	77	70	163	208	67	108	57	88	41	61	87	1292
Records selected	20	20	20	20	20	20	20	20	20	20	20	20	20	20	280
Inaccessible records for 2012	3	7				1		2							13
Inaccessible records for 2013		2						3			2				7
Number of records available for audit	17	13	20	20	20	20	20	17	20	20	20	20	20	20	267
Number of records audited	12	10	13	18	20	20	20	17	14	20	18	20	20	20	242

The audits focused on 2455 consultation reports for the years 2012 and 2013: 754 visits concerning adults and 1701 visits concerning children or adolescents in the 2 years. The number of consultations corresponds to an average of five visits per patient each year (standard deviation: 3.6 to 7.3), all age ranges combined. The 155 children/adolescents and the 87 adults whose records were audited made an average of 5.5 and 4.3 visits per year, respectively. During these 2455 visits, 1855 PFTs were performed to produce source files for FEV1 data (L and %) incorporated into the patient record.

### Local level: Patient records at the CFCs

At the 14 CFCs, patient records were presented in the form of a hard-copy record and an electronic file. The hard-copy record contains examination documents including PFT source files. The electronic record is managed in the Hospital Information System (HIS). At 11 of the 14 CFCs, a software dedicated to cystic fibrosis is used concurrently with the HIS: Five CFCs used the MucoDoméos software, three used the Gulper software, and three used the eMuco software.

#### Discrepancies in the patient records (see Fig. 2)

For the 2455 consultation visits:

- In 67 cases (3%), the consultation report, in which weight and height measurements are recorded, was not found;
- In 45 instances (2%), including 43 instances for adult patients, weight was not recorded in the consultation report; weight anomalies of up to plus or minus 5 kg were identified in 22 cases (1%): these were linked to errors in entry or position of the decimal point;
- In 62 instances (2.5%), including 35 instances (1.5%) for adult patients, height was not recorded in the consultation report; height anomalies of up to 2 cm more or less were identified in 52 cases (2%): these were linked to errors in entry or position of the decimal point;
- In 55 instances (3%), FEV1 results were not recorded in the consultation report; in five instances, the FEV1 value was only that measured after a short-acting bronchodilator was taken; and in 20 instances (1%), the FEV1 value differed from that in the PFT source file.

**Fig. 2:**
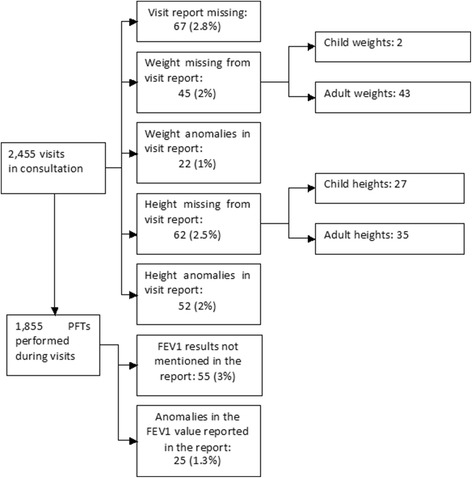
Discrepancies observed in the patient records

Table 3 summarizes the number of discrepancies identified in the records by nature and by CFC.

**Table 3:** Distribution by CFC of the types of discrepancies in the patient records

CFC	1	2	3	4	5	6	7	8	9	10	11	12	13	14	Total	%
No. of consultations audited	168	130	148	162	172	191	173	231	159	219	162	197	151	192	2455	100
Report missing				2	25	1	6	10	15		8				67	2.7
Date missing from report	1	1													2	NS
PFT source file missing	3	1	14	2	6	3	3	2			15				49	2
Weight missing from report	2	1	4		16	18					3	1			45	1.8
Weight anomaly in patient record		1		1	1	18				1					22	0.9
Height missing from report	7	3		7	35				3		6	1			62	2.5
Height anomaly in patient record	2	3	3	2		11	1	13	7	2			4	4	52	2.1
FEV1 missing from report	8	13	7		4				5			18			55	2.2
Discrepancy in FEV1 (L) between report and PFT	2		3		3						3	3	1	5	20	0.8
FEV1 after bronchodilator only in report	3				1			1							5	0.2
Number of PFTs performed	128	49	147	104	173	177	149	144	83	149	147	102	68	236	1855	100
Discrepancy in weight in PFT file	2		16	30	95	97		4	11	2	19	12	18	32	338	18,2
Height anomaly in PFT file			3		2			1					2		8	0,4
Discrepancy in height between PFT file and patient record		30		15	9	80	4	9	6	2	18	13	7	7	200	10,8
Discrepancy in gender in PFTs					1										1	NS

#### Discrepancies in the PFT source files (see Fig. 3)

The organization, equipment, and practices concerning PFTs vary widely from one CFC visited to the next, as illustrated by the description below.

**Fig. 3:**
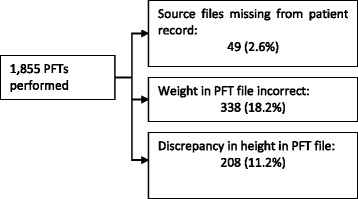
Discrepancies found in the PFT source files

**Organization:** at seven centers, PFTs are performed, and measurements are taken, at the CFC by coordinating nurses (four), physiotherapists (two), or a PFT technician (one); at the other seven CFCs, PFTs are performed in a dedicated department of the hospital by nurses or technicians; one CFC also takes measurements during patient home visits, in which case a physiotherapist takes the measurements; and at the seven hospitals where PFT follow-up is done at the CFC, the annual review takes place in a dedicated department in five cases.

**PFT frequency:** at certain CFCs, PFTs are systematically performed at each visit, i.e. three to four times per year; at other centers, they are performed only once per year for patients in a “good respiratory state”. Mean age at initial PFTs is 4.5 years.

**Equipment:** Forty-four different spirometers are used throughout the 14 CFCs visited. Six are portable devices used in consultations by two CFCs; one is used for home consultations by another CFC; 14 devices are linked to a plethysmography chamber; different brands are represented: Jaeger® (25), Medisoft (nine), EasyOne™ (four), Dyn’R (five), and Spirodoc® (one); the plethysmography chamber is systematically used at one CFC, but never used at four other centers; three CFCs do not have a plethysmography chamber; nine CFCs use both methods; four CFCs systematically use the chamber during the annual review; four use it when it is free; and one mixed CFC uses it for adults only.

**Practice:** patients blow into the spirometer in a seated position at two CFCs and in a standing position at four other CFCs; at eight CFCs, both positions are used depending on patient age, chambers available or patient choice.

**Updated height:** at the 11 pediatric or mixed CFCs, height was updated at each consultation in the spirometry software; at the three adult CFCs, height was not updated at each consultation; and at eight CFCs, height and weight measurements were taken in both PFTs and consultations;

**Standards for calculating FEV1%:** 21 spirometers apply the Zapletal pediatric benchmarks [11] and ECCS/ERS adult benchmarks [12]; seven spirometers only use the Zapletal benchmarks, 14 only use the ECCS/ERS benchmarks, and two centers were unable to specify the benchmarks applied in their spirometer; at three pediatric CFCs, the ECCS/ERS standard was used as a benchmark; and, using the Knudson reference equations [13], the Registry recalculates the FEV1% based on the value in liters transmitted by the center.

**Discrepancies between Patient records and PFT files:** beyond the variability described, the following discrepancies were found:

- Forty-nine PFT source data (2.6%) were missing in the patient records while measurements had been taken and reported in the record; however, it was impossible to audit the value reported;
- Three hundred and thirty-eight discrepancies in weight measurement (18.2%) of up to plus or minus 1 kg were found in the PFTs with respect to the value in the patient records;
- Eight discrepancies on height data of up to plus or minus 2 cm; and
- Two hundred discrepancies on height data of up to plus or minus 1 cm were found between the PFT files and the patient records (11%); these were due to multiple height measurements in a single visit or no update of height in the spirometry data.

It should be noted that:

- Discrepancies on the weight data in the PFT file have no impact on the result of the calculation of FEV1 as a percentage;
- Discrepancies on height (and gender) do have an impact on the result of the calculation of the FEV1 as a percentage.

### National level: results from the control of data transmitted to the registry

The values appearing in the Registry and the values appearing in the patient visit record with the best FEV1 in liters for the year are compared. An *anomaly* is counted each time a difference existed between the values in the Registry and the values in the patient record with the best FEV1 L. When a patient (especially a small child or infant) has not done any PFTs during the year, only the anthropometric data have to be transmitted to the Registry and must be those measured at the last visit for the year. A difference between these values is then counted as an *anomaly*.

Various rules are applied by the CFCs to transmit the spirometry data and the associated anthropometric data to the Registry. The MucoDoméos, Gulper and e-Muco software programs semi-automatically transmitted data to the Registry by selecting the data corresponding to the best FEV1 in liters, for the years 2012 & 2013. Three CFCs did not use software specific to cystic fibrosis and thus transmitted the data selected manually from their Hospital Information System.

Given the variety of procedures for transmitting data to the Registry and the ambiguity of the wording of the rule in the document accompanying the annual Registry questionnaire for the years 2012 and 2013, variability in the selection rules has been observed, causing *anomalies*. The *anomalies* observed are often “massive” as they generally result from the procedure applied at the CFC for all their patients.

The controls showed that:

Nine CFCs transmitted the value in liters corresponding to the best FEV1% for the year to the Registry;

The CFCs using the CF software for semi-automatic transmission generally reported the data corresponding to the best FEV1 in liters for the year;

The weight and height data transmitted to the Registry by the eMuco software are those appearing in the PFT data set, while the primary data measured by the nurse are most often recorded by the healthcare providers at the CFC in the consultation data set;

- Three CFCs transmitted FEV1 values measured after short-acting bronchodilators were taken, in line with their interpretation of the rule for selecting the “best spirometry of the year”;
- At one CFC, in 2013, a replacement staff member entered theoretical FEV1 values by age and sex, instead of patients’ measured values;
- At another CFC, the data transmitted to the Registry corresponded to the last FEV1 for the year in 2012 in accordance with the rule valid up to 2010;
- One CFC did not report FEV1 values in liters in 2012 (only FEV1% value).

In summary, in 110 patient records out of the 242 audited (45%), there were *anomalies* between the FEV1 L appearing in the Registry and the value that would have been expected according to the given selection rule (see Table 4). They mainly relate to the date of the venue not corresponding to the visit at which the best FEV1 value in liters for the year was measured. Other causes of *anomalies* derive from the conditions of FEV1 measurement (transmission of the value after bronchodilator), the absence of data (no transmission of FEV1 L) or an error in the value transmitted (theoretical value). Among those 110 patient records presenting *anomalies*, 33 were children aged 6 to 17 for whom further investigations were made.

**Table 4:** Discrepancies between the patient record data and the Registry data

CFC	1	2	3	4	5	6	7	8	9	10	11	12	13	14	Total	%
No. of records audited	12	10	13	18	20	20	20	17	14	20	18	20	20	20	242	100
No. of records with anomalies	9	3	8	6	10	0	17	4	4	15	10	11	8	5	110	45
Selection of visit date	7		3	6	8		9	5	5	9	8	11	5	2	78	
Weight	4		4	6	11		9	5	4	9	7	11	4	2	76	
Height	3		2	5	1		16	3	5	8	7	8	6	2	66	
FEV1 (L)	4	3	3	6	8		23	5	5	28	7	11	5	4	112	
FEV1 (L) not transmitted	1														1	

### Causes of anomalies concerning the data transmitted to the registry in children

We decided to analyze the causes of the anomalies observed between the Patient Record data (PFT source) and the Registry data in 33 children records (out of the 120 children records controlled) and investigate the potential deviation of FEV1% value resulting from this.

#### Impact of growth on FEV1 L and %

For the 33 children aged 6 to 17, Table 5 shows that:

- For all of them, the visit with the “best FEV1 L” is later in the year than the visit with the “best FEV1%”
- All have grown up between the 2 visits, with height increases of up to 6 cm (average growth = 3.1 cm; median growth = 3.0 cm)
- All have increased their FEV1 L between the 2 visits, in parallel to their height increase, from 0,01 L to 0,49 L (average = 0.1 L; median = 0.06 L)
- All have decreased their FEV1% between the 2 visits, in parallel to their height increase, from – 0.2% to – 11% (average = − 4%; median = − 3%).

**Table 5:** Growth impact on FEV1 L and % for the 33 children presenting a variance btw Patient Record (PFT source) and Registry

33 Patients <18 y.o.			Data of the Visit “Best FEV1 L”					Data of the Visit “Best FEV1%”					Deviation between the 2 Visits		
n°	Birth	F/M	Date visit	Height	Weight	FEV1 L	FEV1%	Date visit	Height	Weight	FEV1 L	FEV1%	Height	FEV1 L	FEV1%
1	2001	F	nov-13	141	29	1,72	89	janv-13	135	25	1,65	96	6	0,07	−7
2	2005	F	oct-13	127	26	1,58	109	mars-13	124	24	1,53	113	3	0,05	−4
3	2005	M	déc-12	125	23	1,47	91	sept-12	122	22	1,37	102	3	0,1	−11
	2005	M	déc-13	130	25	1,54	96	oct-13	128	25	1,51	98	2	0,03	−2
4	2002	F	oct-12	127	23	1,08	72	févr-12	124	20	1,07	76	3	0,01	−4
5	2007	F	nov-13	109	19	1	104	juin-13	108	19	0,99	107	1	0,01	−3
6	1996	F	mai-13	160	57	2,84	84,8	févr-13	160	57,3	2,58	94	0	0,26	−9,2
7	2001	F	juil-13	159	43	2,5	93	janv-13	153	42,3	2,32	96	6	0,18	−3
8	2001	M	oct-12	138,5	31,2	2,12	102,7	juin-12	134	30,4	1,93	110	4,5	0,19	−7,3
9	2002	F	déc-12	130,5	27	1,89	105	juin-12	127	26,6	1,66	114	3,5	0,23	−9
10	2004	F	déc-12	131	26	1,33	77	juin-12	128	25,2	1,2	81	3	0,13	−4
11	2004	F	sept-12	128,5	25,1	1,55	93	juin-12	127	25,5	1,45	104	1,5	0,1	−11
	2004	F	sept-13	133	26,6	1,53	83,7	nov-13	133	27,5	1,39	84	0	0,14	−0,3
12	1996	M	déc-13	177,8	63,5	4,28	108	avr-13	174,5	60,3	4,16	110,5	3,3	0,12	−2,5
13	2001	F	sept-12	126,5	24,6	1,19	83,2	nov-12	125,5	24,8	1,17	83,5	1	0,02	−0,3
14	2001	M	avr-13	143,4	31,5	1,87	87,2	mai-13	142	32	1,86	89,5	1,4	0,01	−2,3
15	2003	M	nov-12	135	27	1,58	87,6	août-12	133,5	25,1	1,55	88,8	1,5	0,03	−1,2
	2003	M	août-13	138,5	28,3	1,58	81,7	janv-13	136	27,6	1,52	82,9	2,5	0,06	−1,2
16	2006	F	sept-12	115	18,4	0,88	79,7	mars-12	112	17,7	0,87	84,3	3	0,01	−4,6
17	2001	M	déc-13	133	27,9	1,08	62,5	avr-13	130	23,9	1,04	64,4	3	0,04	−1,9
18	2009	M	nov-13	109	16,7	0,87	89,5	juil-13	106	15,6	0,84	93,6	3	0,03	−4,1
19	2004	M	oct-13	132	27	1,54	91,3	avr-13	130	27	1,48	91,6	2	0,06	−0,3
20	2008	F	nov-13	111	16	0,89	89,1	janv-13	104,8	15,1	0,8	92,4	6,2	0,09	−3,3
21	1998	F	juin-13	158	56	2,84	107,5	sept-13	157	55	2,81	108,2	1	0,03	−0,7
22	2001	F	déc-12	150	40	1,9	83,1	févr-12	142	36	1,77	89,6	8	0,13	−6,5
	2001	F	avr-13	151,3	46,2	2,15	92,3	janv-13	150	44	2,14	93,4	1,3	0,01	−1,1
23	2007	M	oct-13	113	19	0,98	91,3	févr-13	108	17	0,96	101,4	5	0,02	−10,1
24	2001	F	avr-12	136	28	1,36	77,5	janv-12	133	26,4	1,31	79,4	3	0,05	−1,9
25	2002	F	oct-12	136	30	1,51	86,5	mars-12	132,8	28,4	1,46	88,7	3,2	0,05	−2,2
26	1998	F	nov-13	152,4	37,5	1,76	74,1	mai-13	150,3	35,2	1,7	74,3	2,1	0,06	−0,2
27	1998	M	déc-12	178	57	3,23	81	août-12	175	54	3,1	81,9	3	0,13	−0,9
	1998	M	déc-13	184	64,6	3,69	84,3	mars-13	180	59,5	3,5	85	4	0,19	−0,7
28	2004	M	avr-12	119	1	0,8	73,2	oct-12	122	1	0,67	76,24	3	0,13	−3,04
29	1994	F	nov-12	168	57	3,19	93,4	avr-12	168	53	2,94	94,2	0	0,25	−0,8
30	2001	M	déc-13	150	48	2,02	82,9	janv-13	142	1	1,82	87,3	8	0,2	−4,4
31	1997	M	août-12	160	63	2,94	100,2	janv-12	159	63	2,9	100,6	1	0,04	−0,4
32	1999	M	oct-12	140	35	2,24	112,1	janv-12	136	32	2,21	120,5	4	0,03	−8,4
	1999	M	avr-13	143	39	2,92	115	janv-13	141	37	2,43	119,2	2	0,49	−4,2
33	2005	F	oct-12	123	1	1,21	91,1	janv-12	118	1	1,18	99,8	5	0,03	−8,7
												Average	3,1	0,1	−4,0
												Median	3	0,06	−3

For these 33 children, the choice of selecting the visit with the best FEV1 L or the visit with the best FEV1% does have an impact on the value of FEV1 L transmitted to the Registry (average = 0.1 L; median = 0.06 L).

#### Impact of various standard references for the calculation of the value FEV1%

For the 33 children aged 6 to 17, Table 6 shows that:

- the different selection rules applied were either the best FEV1 L or the best FEV1% or another value corresponding to an undetermined rule. Some standardization appeared when a CF software is used to transmit the data to the Registry;
- the Registry applies the Knudson reference equations to the FEV1 L value transmitted by a CFC: a discrepancy then appeared between the FEV1% in the Registry and the FEV1% in the patient record when local standards used were different from the Knudson reference values, even though the FEV1 L was identical in the 2 files.

**Table 6:** Deviation in FEV1 L and FEV1% btw Patient Record (PFT source – visit when “Best FEV1 L” measured) and Registry for the 33 children among 120 controlled ones

33 Patients <18 y.o.			“Best VEMS L”		“Best VEMS%”		Data in the Registry		Selection & Reference to calculate FEV1%	△ Registry v/s Expected	
n°	Year of Birth	Gender	FEV1 L	FEV1%	FEV1 L	FEV1%	FEV1 L	FEV1%		FEV1 L	FEV1%
1	2001	F	1,72	89,00	1,65	96,00	1.97	100,00	CFC 1: Best FEV1 L and Zapletal Reference	0,25	11,00
2	2005	F	1,58	109,00	1,53	113,00	1.58	111,00		0	2,00
3	2005	H	1,47	91,00	1,37	102,00	1.47	104,00		0	13,00
	2005	H	1,54	96,00	1,51	98,00	1.54	95,00		0	−1,00
4	2002	F	1,08	72,00	1,07	76,00	1.08	74,00		0	2,00
5	2007	F	1,00	104,00	0,99	107,00	1,00	105,00		0	1,00
6	1996	F	2,84	84,80	2,58	94,00	2.43	89,00	CFC 2: Best FEV1%; CFC with Zappletal87; Annual Review in Pnumonology with Jaeger cabin & ECCS93 Quanjer Reference (adult); at Home with Spirodoc	−0,41	4,20
7	2001	F	2,50	93,00	2,32	96,00	2.41	93,00		−0,09	0,00
8	2001	H	2,12	102,70	1,93	110,00	1.93	110,00		−0,19	7,30
9	2002	F	1,89	105,00	1,66	114,00	1.66	114,00		−0,23	9,00
10	2004	F	1,33	77,00	1,20	81,00	1.2	81,00		−0,13	4,00
11	2004	F	1,55	93,00	1,45	104,00	1.55	93,00		0	0,00
	2004	F	1,53	83,70	1,39	84,00	1.39	84,00		−0,14	0,30
12	1996	H	4,28	108,00	4,16	110,50	.	111,00	CFC 3: Transmission of FEV1% only; ECCS93 Quanjer Reference in the spirometer at the CFC	−4,28	3,00
13	2001	F	1,19	83,20	1,17	83,50	.	83,00		−1,19	−0,20
14	2001	H	1,87	87,20	1,86	89,50	.	87,00		−1,87	−0,20
15	2003	H	1,58	87,60	1,55	88,80	.	89,00		−1,58	1,40
	2003	H	1,58	81,70	1,52	82,90	.	82,00		−1,58	0,30
16	2006	F	0,88	79,70	0,87	84,30	.	80,00		−0,88	0,30
17	2001	H	1,08	62,50	1,04	64,40	.	63,00		−1,08	0,50
18	2009	H	0,87	89,50	0,84	93,60	0.91	94,00	CFC 4: Best FEV1 L after bronchodilator and Zapletal Reference	−0,04	4,50
19	2004	H	1,54	91,30	1,48	91,60	1.57	97,00		−0,03	5,70
20	2008	F	0,89	89,10	0,80	92,40	0.89	106,00		0	16,90
21	1998	F	2,84	107,50	2,81	108,20	2.84	108,00		0	0,50
22	2001	F	1,90	83,10	1,77	89,60	1.9	89,00		0	5,90
	2001	F	2,15	92,30	2,14	93,40	2.16	93,00		0,01	0,70
23	2007	H	0,98	91,30	0,96	101,40	0.96	101,00		−0,02	9,70
24	2001	F	1,36	77,50	1,31	79,40	1.38	84,00		0,02	6,50
25	2002	F	1,51	86,50	1,46	88,70	1.52	92,00		0,01	5,50
26	1998	F	1,76	74,10	1,70	74,30	1.85	78,00		0,11	3,90
27	1998	H	3,23	81,00	3,10	81,90	3.33	88,00		0,1	7,00
	1998	H	3,69	84,30	3,50	85,00	3.94	90,00		0,25	5,70
28	2004	H	0,80	73,20	0,67	76,24	0.8	63.9	CFC 5: Random Selection of Best FEV1 L or Best FEV1% maybe depending on the year and Zapletal Reference	0	−9,30
29	1994	F	3,19	93,40	2,94	94,20	3.18	102,00		−0,01	8,60
30	2001	H	2,02	82,90	1,82	87,30	1.82	87.3		−0,2	4,40
31	1997	H	2,94	100,20	2,90	100,60	2.9	100,00		−0,04	−0,20
32	1999	H	2,24	112,10	2,21	120,50	2.21	120,00		−0,03	7,90
	1999	H	2,92	115,00	2,43	119,20	2.69	126,00		−0,27	11,00
33	2005	F	1,21	91,10	1,18	99,80	1.18	99.8		0,03	8,70
									Average	−0,36	4,25
									Median	−0,02	4,00

For the 33 children in the age range of 6–17 years old, the deviation between the FEV1% value appearing in the Registry and the FEV1% value appearing in the Patient Record of the visit with the “Best FEV1 L”, is an average deviation of +4.25% (median deviation = + 4%; min. = −9.30%; max. = + 16.9%).

#### Standardization of data transmitted to the registry with the use of a CF software

The example of 4 pediatric CFCs equipped with the 3 different CF software programed to select the visit at which the “Best FEV1 L” had been measured, shows (Table 7):

- standardization of the data selection in these CFCs
- deviations on FEV1% value remained when the local standard reference in the CFC was different from the Knudson reference value used in the Registry

**Table 7:** Standardization of Data transmitted to the Registry with the use of CF Software

Patients <18 y.o.			“Best VEMS L”		“Best VEMS%”		Data in the Registry		Selection & Reference to calculate FEV1%	△ Registry v/s Expected	
n°	Birth	Gender	FEV1 L	FEV1%	FEV1 L	FEV1%	FEV1 L	FEV1%		FEV1 L	FEV1%
1	1997	M	4,16	108,00	4,11	110,00	4.16	108,00	CFC 1: Best FEV1 L and Zapletal Reference Software Gulper	0,00	0,00
2	2001	F	1,72	89,00	1,65	96,00	1.97	100,00		0,25	11,00
3	2005	F	1,58	109,00	1,53	113,00	1.58	111,00		0	2,00
4	2005	M	1,47	91,00	1,37	102,00	1.47	104,00		0	13,00
	2005	M	1,54	96,00	1,51	98,00	1.54	95,00		0	−1,00
5	2002	F	1,08	72,00	1,07	76,00	1.08	74,00		0	2,00
6	2007	F	1,00	104,00	0,99	107,00	1,00	105,00		0	1,00
35	2000	F	2,43	101,00	2,39	103,00	2.43	101,00	CFC 6: Selection of FEV1 L and Knudson Reference Software Mucodoméos	0	0,00
36	1999	F	2,48	91,00	2,27	92,00	2.48	91,00		0	0,00
	1999	F	2,64	86,00	2,41	87,00	2,64	86,00		0	0,00
37	2012	M	1,21	67	1,18	69	1.21	67	CFC 7: Selection of FEV1 L and Knudson Reference Software e-muco	0	0
38	2004	M	1,69	93	1,65	95	1.69	93	CFC 8: Selection of FEV1 L and Knudson Reference Software: e-muco	0	0
39	2007	F	0,92	89	0,72	93	0.92	89		0	0
40	2003	M	1,76	90	1,57	93	1.76	90		0	0
41	2005	M	1,43	90	1,25	93	1.43	90		0	0
42	2001	F	2,25	108	2,21	112	2.25	108	CFC 9: Selection of FEV1 L and Knudson Reference Software Mucodoméos	0	0
43	2004	F	1,52	107	1,51	114	1.52	108		0	0
	2004	F	1,52	98	1,5	106	1.52	99		0	0

## Discussion

This first on-site quality audit of the data transmitted to the French CF Registry showed a great deal of diversity in terms of organization, information circuits, equipment, and practices concerning taking measurements as well as in the rules applied for selecting the values to be transmitted to the Registry. While *discrepancies* in the recording of each value for weight, height, or FEV1 in liters in the patient records are observed in less than 5% of cases – except for the height in PTF files, discordant in 11% of the 1855 PFTs, *anomalies* between the data appearing in the Registry and the data from the patient records occur for a great number of patients in the sample controlled (45%). The rule of selecting the annual visit when the “best spirometry in the year” was measured, enacted from the 2011 survey, was not homogeneously applied at the 14 CFCs for the years 2012 and 2013, except when the CF Software selected semi-automatically the visit at which the “Best value L” was measured. The use of local standard references instead of the Knudson reference value used in the Patient Registry explains additional deviations in the FEV1% value between the Registry and the patient records when the FEV1 L values matched.

For the 33 children (age range 6–17 years) whose records presented anomalies, an average deviation of the FEV1% value by +4.25% (median = +4%; min. = −9.3%; max. = +16.9%) was observed between the Patient records and the Registry. The impact of the applied selection rule appears to be more critical in this sample as it was observed that respiratory function as reflected by the FEV1% value continuously declined during the 2 years 2012 and 2013 while these children were growing in height, even though their FEV1 value in L had increased.

### Question of reliability of the primary endpoints

The reliability of the primary endpoints used for the research program, FEV1% and BMI, as appearing in the Registry and calculated from the data transmitted by the CFCs was the subject of this on-site audit conducted at 14 CFCs. The discrepancies and anomalies observed may challenge the interpretation of the results for the research program quantitative analysis, which intends to compare the trend of these indicators from 2012 to 2015 between two groups of patients, the PHARE-M group and the Control group. Even though the design of the audit only served the goal of reaching saturation of error causes and not statistical significance, it appeared that on a sample of children and adolescents representing about 33% of the total patients with anomalies, various causes could lead to an average deviation of +4.25% in the value of FEV1% between the Registry and the Patient records (median deviation = + 4%; min. = −9.30%; max. = + 16.9%).

In general, registry data quality, unlike clinical research study data, is rarely audited at the source. However, one intends to use these data for epidemiological studies, phase 4 clinical studies, and care quality improvement follow-up, as well as for national or international comparisons. The European CF Patient Registry Working Group recognizes the current difficulties and limitations in the \interpretation of variations in indicator values across the countries and emphasizes that their transparency may increase their reliability. In France, avenues for improvement have been identified on measurement processes and staff training, organization of data and suitable use of patient information systems, and clear definition and strict application of rules for transmitting data to the Registry.

### Measurement recommendations and staff training

The best practice consists in measuring patient weight and height only once per consultation, at the start, applying the international recommendations for measurement [7]. The results of these measurements should be reported in the PFTs. For adults, the height check is to be done at least once per year, and the weight check at each consultation. A patient’s self-report of their weight and height cannot replace measurement under the required conditions. Multiple measurements by various professionals during a single visit increases the risk of error and cannot compensate for the failure to provide a single measurement done by the required people under the required conditions. The safest way to organize height and weight measurements is to perform them all in one place equipped with devices (height gauges and scales) compliant with the standards and endowed with staff trained in measurement rules and regular monitoring of the devices.

Since the conditions for performing PFTs depend on the patient’s circuit in the hospital, it may be unrealistic to aim to harmonize the organization of PFTs for all CFCs. The most reliable way to organize PFTs would be to ensure that the devices used in different places are compliant with the standard Reference, pediatric or adult, calibrated, and regularly monitored under the responsibility of the PFT department, and are used by trained staff. Knudson reference values should be generalized.

### Organization of data and suitable use of patient information systems

The quality of the organization of the data in the patient record, whatever the format (hard-copy or electronic), is a criterion of the French program of Financial Incentives for Quality Improvement (IFAQ) of patient management. The challenge of the CF electronic patient record is that of taking into account multiple interventions by various professionals in the course of the CF patient’s visit while organizing the data collected in a database such that a given piece of information is recorded in a single structured field. Within the framework of an outpatient visit, the weight and height values measured must be entered just once by a qualified professional, and must be available in read-only real-time mode in the later steps of the patient’s circuit. These electronic records have the advantage of including immediate consistency checks and warnings. In the future, it would be important to conduct a quality audit of software use.

### Clear definition and strict application of rules for transmitting data to the registry

Since 2011, the PRSC has recommended transmitting the data — FEV1 L, weight (kg, g), and height (cm, mm) — corresponding to the “*best spirometry in the year*”. However, this recommendation was ambiguous, as it did not specify if it should be the best value for FEV1 in liters or as a percentage, and in growing individuals, the best value for FEV1 in liters most often does not correspond to the best value as a percentage. In our audit, selection in practice varied by CFC in 2012 and 2013.

The European Registry takes into account the FEV1 L value corresponding to the best FEV1% value for the year [14]. This recommendation was not clearly adhered to in France as the CF patient software programs automatically selected the best FEV1 L value for the year. This issue is to be addressed by the PRSC. Any change in the selection rule would need to be largely explained and implemented in the software used by the CFCs to ensure its application and avoid misinterpretations.

The checks showed that standardization is achieved through automation with software programs selecting suitable data. The annual process of transmitting data to the Registry should be under the responsibility of an identified and trained person at each CFC. An audit of the practices at each site should identify the operations required to check missing or aberrant data and validate the data before transmission.

Just one out of the 14 centers audited did not have any discrepancy in the data. At this center, measurements are taken only once. They are recorded in a software dedicated to cystic fibrosis patients which selects automatically the data to be transmitted to the Registry. The Knudson reference equations are applied in the CFC software. Finally, a person knowledgeable about the instructions is responsible for validating the data before transmitting them to the Registry.

## Conclusions 

In general, registry data quality, unlike clinical research study data, is rarely audited at the source. However, one intends to use these data for epidemiological studies, phase 4 clinical studies, and care quality improvement follow-up, as well as for national or international comparisons. The European CF Patient Registry Working Group recognizes the current difficulties and limitations in the \interpretation of variations in indicator values across the countries and emphasizes that their transparency may increase their reliability. This first on-site quality audit of the data transmitted to the French CF Registry pointed out variability in the measurement process at the CFCs. The rule for selecting the data for the Registry was applied differently at some CFCs, and various local References for the FEV1% calculation were used. Avenues for improvement have been identified. They include measurement processes staff training, organization of data, suitable use of patient information systems, clear definition and strict application of rules for transmitting data to the Registry.
